# Sleep–Wake Disturbances in Pediatric Traumatic Brain Injury: A Systematic Review and Meta‐Analysis of Interventions

**DOI:** 10.1002/brb3.71556

**Published:** 2026-09-21

**Authors:** K. Lofitou, E. P. Kouki, C. Gavriel, H. Achilleos, V. Themistokleous, P. Bargiotas, J. Haarbauer‐Krupa, E. Paraskevopoulos, F. Constantinidou

**Affiliations:** ^1^ Center for Applied Neuroscience University of Cyprus Nicosia Cyprus; ^2^ Department of Psychology University of Cyprus Nicosia Cyprus; ^3^ Department of Psychology University of Limassol Limassol Cyprus; ^4^ Department of Pediatric Accident & Emergency, Nicosia General Hospital State Health Services Organization Nicosia Cyprus; ^5^ Medical School University of Cyprus Nicosia Cyprus; ^6^ Department of Neurology Nicosia General Hospital Nicosia Cyprus; ^7^ Department of Pediatrics Emory University School of Medicine Atlanta Georgia USA

**Keywords:** cognitive behavioral therapy for insomnia, meta‐analysis, pediatric traumatic brain injury, sleep interventions, sleep–wake disturbances

## Abstract

**Background:**

Sleep–wake disturbances (SWD) are frequent and persistent complications following pediatric traumatic brain injury (TBI), negatively affecting cognitive, emotional, and academic outcomes. This systematic review and meta‐analysis aimed to evaluate the effectiveness of behavioral, pharmacological, and emerging interventions for SWD in children and adolescents with mild to severe TBI and examined related sleep outcomes and functional measures.

**Method:**

Following PRISMA guidelines, a systematic search was conducted across PubMed, Web of Science, and ScienceDirect for studies published between 1998 and 2025. Nineteen studies (*N* = 1113) met inclusion criteria, encompassing cognitive behavioral therapy for insomnia (CBT‐I), sleep hygiene, mindfulness, pharmacological treatments (melatonin, branched‐chain amino acids), physical activity, and family‐based interventions. Meta‐analytic models using random effects were employed to calculate pooled standardized mean differences and mean raw scores across sleep, cognitive, and quality‐of‐life outcomes.

**Results:**

CBT‐I demonstrated efficacy in reducing persistent post‐concussion symptoms and improving subjective sleep outcomes, although heterogeneity varied across outcomes. In contrast, melatonin did not produce statistically significant pooled effects on persistent post‐concussion symptoms or cognitive outcomes, and treatment effects varied across dosage groups. Few studies examined mindfulness or educational outcomes, revealing key research gaps. Discrepancies between objective (actigraphy) and subjective (self‐report) measures emphasized the need for multi‐method sleep assessment.

**Conclusion:**

CBT‐I is the most consistently effective intervention for sleep problems following pediatric TBI. Melatonin showed mixed and nonsignificant pooled effects, indicating that its role in pediatric TBI recovery requires further investigation. Future research should focus on personalized, multidisciplinary treatment models integrating behavioral, pharmacological, and rehabilitative approaches to optimize long‐term neurocognitive and educational outcomes.

## Graphical abstract

This systematic review and meta‐analysis of 19 studies (*N* = 1113) examined interventions for sleep–wake disturbances following pediatric traumatic brain injury. Cognitive behavioral therapy for insomnia (CBT‐I) consistently improved sleep quality and reduced post‐concussion symptoms, while pharmacological treatments such as melatonin showed mixed and nonsignificant pooled effects. Mindfulness and physical activity approaches demonstrated preliminary promise but remain underexplored.

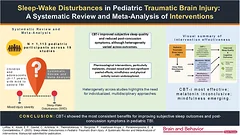

## 1 Introduction

Traumatic brain injury (TBI) is the leading cause of disability and death in children and adolescents (Galland et al. 2012), characterized by abrupt disruption of brain function or damage to brain tissue due to external force. In pediatric populations, healthcare providers often prioritize acute, visible consequences of TBI, with sleep problems frequently under‐recognized and undertreated (Weber et al. 2013). Yet, sleep–wake disturbances (SWD) are common sequelae of pediatric TBI, reflecting injury‐related disruptions in neural systems that regulate sleep and wakefulness (Gagner et al. 2015; Williams et al. 2020). In this review, the term SWD describes the overall construct encompassing insomnia, excessive sleepiness, circadian rhythm disorders, and other post‐TBI sleep–wake disruptions. The term sleep problems refers specifically to participant‐ or caregiver‐reported difficulties (subjective sleep problems) such as problems initiating or maintaining sleep or poor perceived restorative sleep. The term sleep outcomes refers to objectively or subjectively measured endpoints (for example, actigraphy sleep efficiency, total sleep time, or Insomnia Severity Index [ISI] scores).

These disturbances, which can persist for years after injury, are typically more severe and enduring than developmental sleep issues in children without TBI (Dietch and Furst 2020; Moore et al. 2021). SWD in pediatric TBI include insomnia, excessive sleepiness, circadian rhythm disorders, sleep‐related seizures, and breathing deficits. Insomnia is defined by inadequate sleep quantity or quality despite opportunities for rest, with consequences such as impaired daytime functioning (American Psychiatric Association 2013; American Academy of Sleep Medicine 2014). Sleep deficits in this population are strongly linked to poorer cognition, emotional dysregulation, and reduced quality of life (Dietch and Furst 2020; Reynolds et al. 2023).

The developmental stage of adolescence further compounds vulnerability: Poor sleep in this period reduces reaction time, impairs emotional regulation, increases risk‐taking, and negatively impacts school performance (Beebe 2011; Shochat et al. 2014). Given the reciprocal link between sleep disruption and mood disorders, addressing SWD is central to neuropsychological well‐being and recovery following TBI (Reynolds et al. 2023).

Despite the clear importance of sleep for recovery, insomnia and related disturbances often remain undetected in pediatric TBI patients unless raised as a primary complaint (Weber et al. 2013). Prevalence estimates of insomnia in typically developing children range from 25%–40% (Medalie et al. 2021), increasing to 40% in adolescents (Chung et al. 2014). Children aged 0–4 years and adolescents aged 15–19 years face the highest concussion risk, with males overrepresented (Ludwig et al. 2020). Given these patterns, early identification and effective intervention for SWD in TBI are essential (Lah and Cao 2024).

Treatment approaches can be direct or indirect: Addressing sleep may enhance recovery from the underlying injury, while TBI rehabilitation may indirectly benefit sleep (Galland et al. 2012). Evidence shows that treating SWD in children with TBI improves cognition and behavioral regulation, core areas of injury‐related impairment (Dietch and Furst 2020; Moore et al. 2021). The importance of sleep in brain injury recovery implies that managing sleep problems in neurological conditions (i.e., TBI) may play a vital role in supporting optimal recovery (Weber et al. 2013). Psychological and behavioral interventions are the endorsed primary treatments for insomnia, as endorsed by the American Academy of Sleep Medicine (Ree et al. 2017; Jacobs et al. 2004) and the Australian Sleep Association (Ree et al. 2017). These interventions provide clinically meaningful and sustained benefits, often outperforming pharmacological treatments (Jacobs et al. 2004).

Caregiver involvement is the key to identifying SWD, especially in young children with difficulties sleeping alone, initiating sleep, or maintaining sleep (Williamson et al. 2019). Behavioral strategies such as extinction methods, consistent bedtime routines, and reinforcement can effectively address bedtime refusal and night waking (Tikotzky and Sadeh 2010; Owens and Mindell 2011).

### 1.1 The Present Study

Existing systematic reviews have emphasized the prevalence and clinical characteristics of SWD in pediatric TBI (Botchway et al. 2019; Séguin et al. 2022). These studies highlight SWD as frequent, long‐lasting, and impactful but provide little guidance on effective interventions. A major limitation of the literature is its narrow focus, often excluding interventions where sleep is a secondary outcome (Galland et al. 2012). Consequently, the evidence based on comprehensive treatment strategies for this population remains fragmented.

As Séguin et al. (2022) concluded, despite the significant burden of SWD on pediatric recovery, interventions remain underexplored. For example, while pharmacological options (e.g., melatonin), behavioral therapies (cognitive behavioral therapy for insomnia [CBT‐I], sleep hygiene), mindfulness, and family‐based approaches have been studied in adolescents, little is known about their effectiveness in younger children or across TBI severities. Addressing this gap requires a systematic and comparative evaluation of existing approaches. To respond to this gap, the current systematic review and meta‐analysis evaluates the effectiveness of diverse sleep interventions in children with mild to severe TBI. The study examines cognitive‐behavioral, pharmacological, mindfulness, psychoeducational, and family‐based interventions across sleep, cognitive, and educational outcomes.

Our hypotheses are the following:

**RQ1**. *Sleep interventions improve sleep quality and reduce SWD severity in pediatric TBI. Given the persistence of SWD post‐injury* (Gagner et al. 2015; Séguin et al. 2022 *), we expect moderate to significant improvements, moderated by intervention type and participant profile*.
**RQ2**. *Non‐pharmacological approaches (CBT‐I, sleep hygiene) yield more sustained sleep benefits than pharmacological treatments, consistent with prior evidence (*Galland et al. 2012; Dietch and Furst 2020; Moore et al. 2021; Ree et al. 2017; Qaseem et al. 2016).
**RQ3**. *Improvements in sleep outcomes positively influence cognition, emotional regulation, and educational performance, reflecting the strong link between sleep and neuropsychological health (*Reynolds et al. 2023; Beebe 2011; Shochat et al. 2014 *)*.

## 2 Methods

The present systematic review and meta‐analysis followed the Preferred Reporting Items for Systematic Reviews and Meta‐Analyses (PRISMA) protocols (Moher et al. 2015; Page et al. 2021) and the *Cochrane Handbook* (Higgins and Thomas 2022). The study protocol was registered in the International Prospective Register of Systematic Reviews (PROSPERO) prior to the start of the study (available at: https://www.crd.york.ac.uk/prospero/export_details_pdf.php).

### 2.1 Study Selection

The study selection process was guided by the following inclusion and exclusion criteria:

1. English‐language peer‐reviewed publications from 1998 to 2025.
2. Participants aged 0–17 years diagnosed with mild, moderate, or severe TBI.
3. Interventions including CBT‐I, sleep hygiene, mindfulness, pharmacological treatments (melatonin or related sleep medications), physical activity, or family‐based approaches.
4. Comparisons with typically developing children, children/adolescents with TBI‐related insomnia, young adults with the same diagnosis, treatment as usual, or placebo.
5. Outcomes assessing sleep quality, total sleep time, sleep efficiency, cognitive functioning, or educational performance, measured with either objective (e.g., actigraphy) or subjective tools (e.g., sleep diaries, validated questionnaires).
6. Study designs including randomized controlled trials (RCTs), observational studies, case studies, or study protocols.

The PRISMA 2020 checklist, the full search strategies for all databases, and the quality assessment are available in the Open Science Framework (OSF) at: https://osf.io/4jhmr/?view_only=967c2c0a3201490eb722226adfbb8e6a.

### 2.2 Study Reviewers

**FIGURE 1:**
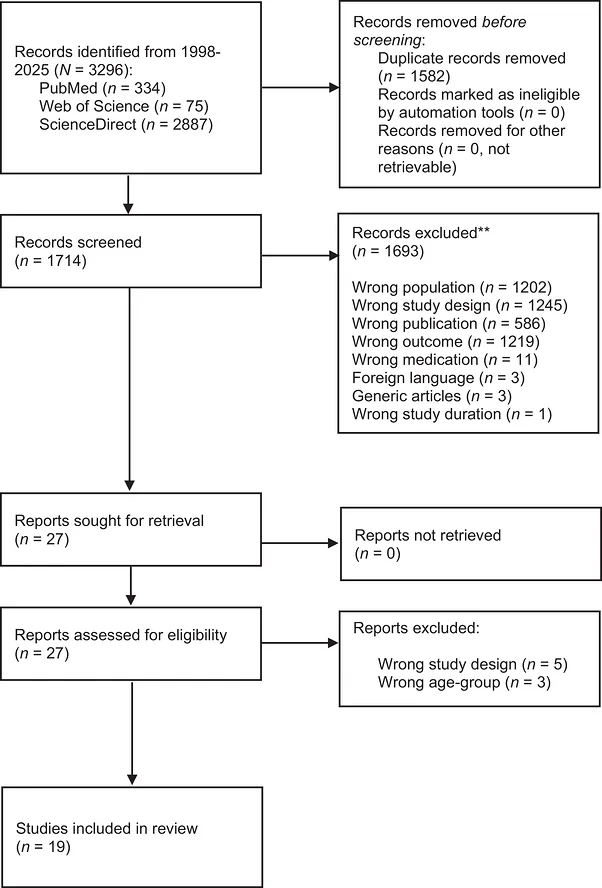
PRISMA flow chart of the study selection process.

Two independent groups of reviewers screened the titles and abstracts identified in the systematic search. Reviewer Group 1 (V. Themistokleous [V. T.] and C. Gavriel [C. G.]) independently screened all titles and abstracts, while Reviewer Group 2 (E. P. Kouki [E. P. K.] and K. Lofitou [K. L.]) conducted an independent parallel review. Any discrepancies between the two groups were resolved through consensus. Reviewers within each group were not blinded to their respective searches. After abstract screening and study identification, Reviewer Group 1 (V. T. and C. G.) and Reviewer Group 2 (E. P. K. and K. L.) independently assessed full‐text articles for eligibility based on the inclusion and exclusion criteria. The two groups discussed disagreements during this stage and examined any unresolved discrepancies. After selecting eligible studies, a manual review of the reference lists from the included articles was conducted to detect additional relevant studies not captured in the initial search. To enhance comprehensiveness, included studies were entered into Google Scholar to track their citations. A final search update was conducted in January 2025 to identify any newly published studies that met the eligibility criteria. This update included an automated alert system from the indexed databases (PubMed, Web of Science, and ScienceDirect) that notified the authors of any new publications meeting the predefined criteria prior to manuscript submission. No additional eligible studies were identified. The PRISMA flow chart (Figure 1) outlines the study selection process. The most common reasons for exclusion were the wrong population (*n* = 1202), wrong study design (*n* = 1245), wrong outcome (*n* = 1219), or wrong publication (*n* = 586; i.e., reviews, meta‐analyses, editorials). The exclusion categories were not mutually exclusive, as some records met more than one exclusion criterion during title and abstract screening. Therefore, the reported frequencies represent overlapping exclusion categories rather than independent counts. Reviewers were not blinded to the journal titles, study authors, or institutional affiliations. Cohen's Kappa was calculated to measure inter‐rater agreement, which revealed satisfactory agreement between the two reviewer groups (Cohen's *k* = 0.71). Nineteen studies were eligible for full‐text screening, and a Cohen's Kappa analysis revealed high agreement between the two reviewer groups (Cohen's *k* = 0.86).

### 2.3 Meta‐Analytic Approach

A quantitative synthesis (meta‐analysis) was conducted to assess the effectiveness of sleep interventions in children with TBI across several outcome domains. Meta‐analytic procedures were applied when studies reported sufficient quantitative data (e.g., means, standard deviations, and sample sizes) for comparable outcomes. Pooled effect sizes were calculated for the following domains: post‐concussion symptomatology, sleep quality and efficiency, cognitive functioning, and overall quality of life. For continuous outcomes, either standardized mean differences (SMDs) or mean raw scores (MRAWs) were computed, depending on the consistency of reporting across studies. SMDs were used when comparable outcomes were assessed using different measurement scales, whereas MRAWs were retained when studies reported outcomes on the same scale or in pre‐post analyses without sufficient data to reliably standardize effect sizes. A random‐effects model using the inverse variance method was applied to account for anticipated heterogeneity due to differences in study design, intervention type, participant characteristics, and measurement tools. All pooled effects were reported with 95% confidence intervals (CIs). Heterogeneity was assessed using the *I* ^2^ statistic, with thresholds of 25%, 50%, and 75% indicating low, moderate, and high heterogeneity, respectively. Subgroup analyses were conducted where feasible to explore sources of heterogeneity, including intervention type (e.g., CBT‐I vs. melatonin), melatonin dosage (3 vs. 10 mg), and measurement method (objective vs. subjective sleep outcomes). Studies contributing to each pooled analysis were inspected for consistency in the direction and magnitude of effects. Forest plots were visually examined to identify potential dispersion and outliers. Given the limited number of studies per outcome domain, formal tests for publication bias (e.g., Egger's regression, funnel plot asymmetry) were not performed, except where at least 10 studies were available for a given outcome.

## 3 Results

The PRISMA flow chart in Figure 1 summarizes the results from database searches and screening procedures. A total of 3296 studies emerged from the main search across databases and were screened at the title and abstract level. Twenty‐four studies were eligible according to the inclusion criteria. After a manual search, three additional studies were added. Of the 27 total studies, all were retrieved and assessed for eligibility. After quality assessment, 19 studies were included for data extraction.

### 3.1 Study Characteristics and Sleep Outcomes

The characteristics of the included studies (*N* = 19) are summarized in Table 1, including participant demographics, TBI severity, time since injury (TSI), comorbidities, and intervention types. Study designs encompassed CBT‐I (*n* = 6), broader cognitive‐behavioral or multidisciplinary psychological interventions (*n* = 2), pharmacological treatments (*n* = 4), physical activity (*n* = 3), psychoeducation or self‐management (*n* = 3), and mindfulness (*n* = 1). Sleep outcomes were assessed using objective tools (actigraphy; *n* = 6) and subjective measures (ISI, sleep diaries; *n* = 8). Objective (actigraphy) outcomes included sleep efficiency and total sleep time, whereas subjective (self‐report/sleep diaries) measures included ISI scores, sleep diaries, and reported sleep onset latency. Further study‐specific information, including screening tools, intervention protocols, and outcomes, is provided in Table 2.

**TABLE 1:** Participant demographics and intervention types.

	Severity type	Age at injury	Time post‐injury	Gender	Comorbidities	Intervention type	Number of participants
1. Barlow et al. (2014)	Mild	13–18	Within 30 days	n/r	n/r	Placebo, melatonin 3 mg, melatonin 10 mg	n/r
2. Lah et al. (2021)	GCS = 6–14	11–13	n/r	1F, 4M	Learning difficulties, memory difficulties, speech and social communication skills difficulties, social anxiety, family relationship difficulties	CBT‐I	5
3. Tomfohr‐Madsen et al. (2020)	PPCS and GCS ≥ 13, 30 min post‐injury, LOC < 30 min, posttraumatic amnesia < 24 h	12–18 years, mean 15.0 (SD: 1.4)	1–12 months, mean 15.1 (SD: 9.2) weeks	18F, 6 M (9F, 3 M in each group)	n/r	CBT‐I	24
4. Moore et al. (2021)	n/a	n/a	n/a	n/a	n/a	CBT‐I	15
5. McNally et al. (2018)	GCS ≥ 13–15	Mean = 15.90 (SD: 2.00)	3 weeks to 2 years	21F, 10M	ADHD, anxiety or mood disorder, ODD, substance abuse	CBT‐I	31
6. Simpson et al. (2021)	n/r	8–17	1–12 months	22F, 8M	Anxiety and mood disorders (also measured as outcomes)	CBT‐I	30
7. Hunt et al. (2016)	n/r	5–18	n/r	n/r	Not reported	Educational program	87
8. Smulligan et al. (2023)	PCSI = 9	13–18	Within 14 days	42F, 30M	ADHD, previous TBI, headaches and migraines	Physical activity	55
9. Barlow et al. (2020)	PSCI ≥ 10 increase post‐injury	8–18 years, mean age 14.0 (SD: 2.6) years	4–6 weeks	52F, 20M	NCI, anxiety, depression, headaches, QRHL scores are similar.	Placebo, melatonin 3 mg, melatonin 10 mg	72
10. Barlow et al. (2021)	PPCS ≥ 10 point PCSI increase	8–18 years, mean age 13.8 (SD: 2.6) years	4–6 weeks	57F, 42M	Previous concussion, migraine, ADHD, learning support, previous counselor, PCSI‐Y score pre‐injury. Excluded significant medical/psych histories. Excluded previous concussion (3 months).	Placebo, melatonin 3 mg, melatonin 10 mg	99
11. Dobney et al. (2017)	PCSS median = 18	Mean = 14.1 (SD: 2.3)	Mean = 40 days (SD: 7.4)	135F, 142M	n/r	Physical activity	277
12. Anderson et al. (2021)	GCS ≥ 13	8–18	Less than 17 days	n/r	n/r	Multimodal	n/r
13. Yang et al. (2019)	n/r	7–17	Within 72 h	n/r	n/r	Active rest	n/r
14. Ledoux et al. (2024)	GCS ≥ 13	n/r	Within 7 days	n/r	n/r	Mindfulness	n/r
15. Shore et al. (2022)	n/r	n/r	n/r	n/r	n/r	Physical activity (virtual)	n/r
16. Corwin et al. (2024)	Mild TBI	11–34	2–2.5 days	18F, 20M	n/r	BCAA	38
17. Mazzone et al. (2024)	n/a	n/a	n/a	n/a	n/a	n/a	11
18. McNally et al. (2024)	CCC = 40	16	15 days	1F	n/r	CBT‐I	1
19. McCarty et al. (2024)	Three or more concussion symptoms	n/r	1–12 months	n/r	n/r	CBT (concussion‐focused), parenting skills training, care management	368

Abbreviations: ADHD = attention‐deficit/hyperactivity disorder, BCAA = branched‐chain amino acids, CBT‐I = cognitive behavioral therapy for insomnia, CCC = Concussion Clinical Criteria, F = female, GCS = Glasgow Coma Scale, LOC = loss of consciousness, M = male, n/a = not applicable, n/r = not reported, NCI = neurocognitive impairment, ODD = oppositional defiant disorder, PCSI = Post‐Concussion Symptom Inventory, PCSI‐Y = PCSI–Youth version, PCSS = Post‐Concussion Symptom Scale, PPCS = persistent post‐concussion symptoms, QRHL = quality of recovery health literacy, SD = standard deviation, TBI = traumatic brain injury.

**TABLE 2:** Study characteristics, sleep outcomes, and intervention details.

Authors	No. of participants × type of group	Time since injury (TSI) at inclusion	Etiology of TBI	Main aim	Screening tools	Occurrence of SWD	Relation between SWD with school and cognitive functioning	Intervention
Barlow et al. (2014)	Protocol: *N* = 99 (3 mg melatonin = N1, 10 mg melatonin = N2, placebo = N3; 1:1:1)	30 days post‐injury	Falls and sport‐related injuries.	(a) Examine the effect of different dosages of sublingual melatonin on PPCS in children. (b) Assess if there is a dose‐response relationship and whether the treatment effects are independent of changes in sleep.	PCSI‐P and PCSI‐Y, CHQ, Behaviour Assessment System for Children, BRIEF, CNS‐Vital Signs, actigraphy	Protocol	Protocol	Administering 3 or 10 mg of sublingual melatonin daily for 28 days
Lah et al. (2021)	*N* = 24, randomized (*n* = 7), allocated to 7‐day baseline and CBT‐I: 5, allocated to 14‐day baseline and CBT‐I: 2, final analysis: two from each group	Range from 4 months to 11 years.	Falls and non‐accidental injuries	(a) Assess the feasibility of delivering CBT‐I to adolescents with TBI who are also receiving standard treatment. (b) Gather pilot data on changes in sleep and fatigue following the intervention.	SDSC, clinical interview using a modified version of the RDCI, ISI, actigraphy watches	Inclusion criteria: T‐score > 55 on the SDSC, met clinical criteria for insomnia, and a score ≥ 8 on the ISI. Three‐fourths of the adolescents who completed treatment showed improvements in sleep.	Improvement in daytime and cognitive fatigue. No specific relation with school performance.	A manualized CBT‐I program aimed at adolescents with TBI, focusing on establishing a consistent sleep schedule, strengthening the bed–sleep association, and reducing insomnia‐related factors.
Tomfohr‐Madsen et al. (2020)	*N* = 78 (total randomized participants: 24, CBT‐I group: 12, waitlist group: 12)	> 1 month; < 12 months post‐injury	Not explicitly stated	(a) To assess if CBT‐I reduces insomnia and improves sleep outcomes in adolescents with prolonged concussion recovery, compared to TAU. (b) Examine the effects on depression, anxiety, and PPCS.	ISI baseline; ISI online daily sleep diaries; 24‐h sleep patterns interview; PSQI; Dysfunctional Beliefs and Attitudes about Sleep Scale; PROMIS Anxiety and Depression; HBI; sleep diary	CBT‐I led to significant insomnia reduction, improved sleep quality and beliefs, over 1 h more sleep, better efficiency, and increased total sleep time, especially at 1‐month follow‐up.	They do not directly address the relationship between SWD and school or cognitive performance.	CBT‐I: weekly in‐person sessions with psychoeducation, relaxation techniques, sleep consolidation, stimulus control, cognitive restructuring, and relapse prevention.
Moore et al. (2021)	*N* = 15 clinicians (clinical psychologists, neuropsychologists, psychologists, social workers, etc.)		Not explicitly stated	Assessment of the acceptability, feasibility, and usability of a CBT‐I program adapted for children aged 6–12 years with TBI from the perspective of allied health clinicians.	Online survey for clinicians. Within the program: sleep diary and manuals for parents, children, and therapists (Excel file).			Manualized CBT‐I program, adapted for children aged 6–12 with msTBI, aiming to establish consistent sleep patterns and reduce insomnia.
McNally et al. (2017)	*N* = 31	Between 23 days to 2 years; median TSI: 3 months	Sports‐related injuries, motor vehicle accidents, falls, and other causes.	Evaluate the effectiveness of a treatment model for children and adolescents with persistent symptoms following a concussion, particularly those who did not respond to first‐line concussion services.	GCS, SCAT‐3, modSCAT‐3, PedsQL. History questionnaire and neuroimaging reports, CT or MRI were also collected.	SWD wasn't the primary focus, sleep regulation was noted as important for managing PPCS.	The treatment significantly improved school attendance and overall quality of life but there is no direct link between SWD and school and cognitive functioning.	45–60‐min long sessions with a therapist, guided by a biopsychosocial framework. Sessions included psychoeducation, activity and sleep scheduling, relaxation training, and cognitive restructuring.
Simpson et al. (2021)	*N* = 30	Concussion occurred 1–12 months prior to enrollment, with a median TSI of 64 days.	Sports‐related injuries, motor vehicle accidents, falls, and other causes.	Evaluation of a brief CBT intervention for children with persistent PPCS, targeting symptom reduction, quality of life, sleep, school attendance, and activity levels.	SCAT‐3, HBI, PedsQL 4.0 Generic Core Scales and RCADS. Information about injury‐related variables was sustained through HRQOL and PPCS.	Of the 21 participants who initially took at least 30 min to fall asleep, 17 (81%) improved to under 30 min, 3 remained in the 30–60 min category, and 1 participant increased to over 60 min.	Improved school attendance but no direct link between SWD and school and cognitive functioning.	CBT‐based intervention for children and adolescents with PPCS.
Hunt et al. (2016)	62 parents/caregivers and 25 adolescents	4–8 weeks	Not mentioned	To develop, implement, and evaluate the feasibility of an evidence‐informed self‐management education program designed for children recovering from concussion.	Anonymous tools (i.e., demographics, feasibility data, knowledge uptake).	While the occurrence of SWD is not specifically measured, sleep hygiene is recognized as part of concussion recovery.	No direct link	A self‐management education program for children recovering from concussions.
Smulligan et al. (2023)	*N* = 55, concussion = 34 (*n* = 10 in the early PA group and *n* = 24 in the no PA group), uninjured = 21 (control)	Up to 14 days	Sports accidents	Assess the association between early PA and post‐concussion sleep quality, dizziness, and postural stability in adolescents.	PCSI, PSQI, DHI, Timed Tandem Gait Test	The no PA group had significantly poorer sleep quality, while the early PA group did not, indicating that early PA may have improved sleep quality.	The study does not directly address the relationship between SWD and cognitive or school performance.	Assessing the impact of early physical activity following concussion.
Barlow et al. (2021)	*N* = 72 (placebo: *n* = 22, melatonin 3 mg: *n* = 25, melatonin 10 mg: *n* = 25)	4–6 weeks	Not explicitly stated; however, it mentioned that sleep problems are common across the spectrum of TBI, suggesting that the study may have included participants with various causes of TBI.	To evaluate the efficacy of treatment with melatonin for sleep disturbance in youth with PPCS following mTBI.	PCSI, BASC‐2, actigraphy.	The study found that melatonin significantly improved SWD in children and adolescents with PPCS after mTBI, decreasing subjective (self‐report) sleep problems, increasing total sleep time and efficiency, and reducing depression symptoms, but it had no significant effect on pain, anxiety, or cognitive function.	The study found that melatonin improved sleep in children and adolescents with PPCS following mTBI. Improved sleep may have positively impacted school performance and cognitive function, though further research is needed. Also, showed the importance of addressing SWD, as they significantly affect quality of life and academic success.	The study used a randomized controlled trial to assess melatonin's efficacy in treating SWD in children and adolescents with PPCS after mTBI. Participants were randomly assigned to a placebo, 3 mg, or 10 mg melatonin group, with sublingual administration for 28 days. Sleep‐related problems were measured using the PCSI, along with objective sleep‐activity data.
Barlow et al. (2020)	*N* = 99 (placebo: *n* = 33, melatonin 3 mg: *n* = 33, melatonin 10 mg: *n* = 33)	4–6 weeks	Motor vehicle accident (*n* = 4), sports (*n =* 23), fall (*n* = 2), witnessed (*n =* 32)	To determine if treatment with melatonin improves PPCS after mTBI and concussion in youth.	PCSI, TOMM, BASC‐2, BRIEF, CNS Vital Signs, actigraphy.	No significant group differences	The study suggests SWD may indirectly affect school and cognitive functioning in children with PPCS, but melatonin had no significant effect on SWD. Further research is needed to confirm the link between SWD and cognitive performance.	Pharmacological (melatonin)
Dobney et al. (2017)	*N* = 277	3–4 weeks post‐injury.	Sports‐related concussions were the most commonly reported in the sample (*n* = 217).	(a) To estimate changes in PPCS due to participating in the AR program in children and adolescents. (b) To examine the influence of sex on PPCS evolution.	PCSS from the SCAT 2 and 3.	Ιmproved PPCS severity compared to pre‐intervention, across all symptom clusters, including physical, cognitive, emotional, and sleep‐related symptoms.	Τhe study did not directly measure the relationship between SWD and cognitive functioning.	AR program
Anderson et al. (2021)	*N* = 216, (CE: *n* = 108, UC: *n* = 108)	< 17 days	n/r	To assess the effectiveness of the "Concussion Essentials (CE)" intervention for treating PPCS in children and adolescents.	PCSI, SDQ, GCS.	Difficulty falling asleep, trouble staying asleep, and frequent night awakenings.	More pronounced SWD affected cognition (e.g., attention, memory, and processing speed), keeping up with academic demands, and increased school absences.	CBT‐based intervention.
Ledoux et al. (2024)	*N* = 70 (MBI: *n* = 35, controls: *n* = 35)	7 days	n/r	To assess the feasibility of a digital therapeutic (DTx) MBI in adolescents.	Predicting and Preventing PPCS in Pediatrics (5P), PedsQL, Multidimensional Fatigue Scale PedsQL, HBI, PCSI, SEQ‐C, NIH Toolbox Cognitive Battery, CD‐RISC‐10, GAD‐7, CES‐DC, and CAMM.	n/r	n/r	A digital MBI.
Shore et al. (2022)	Not reported	2 weeks	Motor vehicle accidents, falls, sports‐related injuries.	To develop the Tele‐AR intervention for youth with concussion.	PCSI, PCERT, Wong–Baker FACES pain rating, VAS.	No data collection yet.	No data collection yet.	Tele‐AR intervention (teleconferencing‐based intervention including aerobic exercise, coordination drills, and comprehensive concussion education).
Corwin et al. (2024)	*N* = 38 (placebo group, *n* = 8, 15 g BCAA group: *n* = 8, 30 g BCAA group: *n* = 5, 45 g BCAA group: *n* = 9, 54 g BCAA group: *n* = 8).	72 h	Motor vehicle accidents, falls, sports‐related injuries.	To determine the efficacy, tolerability, and safety of varied doses of oral BCAA supplementation in a group of concussed adolescents and young adults.	CogSport/Axon Sports, CCAT, actigraphy, self‐reports on symptom assessment, physical and cognitive activity.	Highest BCAA dose group (54 g/day): higher sleep efficiency (93.2% vs. 91.4%) and longer total sleep time (536.7 min vs. 468.1 min) than the placebo group.	No significant changes in cognition.	Pharmacological (branched‐chain amino acids [BCAAs])
Mazzone et al. (2024)	11 clinicians	n/r	n/r	To explore how interdisciplinary rehabilitation clinicians recognize, assess, and manage fatigue in children (0–18 years) with ABI and the facilitators and barriers to assessing and managing fatigue in pediatric ABI rehabilitation.	PedsQL MFS	n/r	n/r	n/s

Abbreviations: ABI = acquired brain injury, AR = active rehabilitation, *BASC‐2* = *Behavior Assessment System for Children Second Edition*, BCAA = branched‐chain amino acids, BRIEF = Behavior Rating Inventory of Executive Function, CAMM = Child and Adolescent Mindfulness Measure, CBT‐I = cognitive behavioral therapy for insomnia, CCAT = Computerized Cognitive Assessment Tool, CD‐RISC‐10 = Connor‐Davidson Resilience Scale–10 item, CE = concussion essentials, CHQ = Child Health Questionnaire, CNS = central nervous system, DHI = Dizziness Handicap Inventory, DTx = digital therapeutic, F = female, GAD‐7 = Generalized Anxiety Disorder 7‐item scale, GCS = Glasgow Coma Scale, HBI = Health and Behavior Inventory, HRQOL = health‐related quality of life, ISI = Insomnia Severity Index, M = male, MBI = mindfulness‐based intervention, MRI = magnetic resonance imaging, mTBI = mild traumatic brain injury, n/a = not applicable, n/r = not reported, n/s = not specified, PA = physical activity, PCERT = Pictorial Children's Effort Rating Table, PCSI = Post‐Concussion Symptom Inventory, PCSI‐P = PCSI–Parent version, PCSI‐Y = PCSI–Youth version, PCSS = Post‐Concussion Symptom Scale, PedsQL = Pediatric Quality of Life Inventory, PedsQL MFS = Pediatric Quality of Life Multidimensional Fatigue Scale, PPCS = persistent post‐concussion symptoms, PROMIS = Patient‐Reported Outcomes Measurement Information System, PSQI = Pittsburgh Sleep Quality Index, RCADS = Revised Children's Anxiety and Depression Scale, RDCI = Research Diagnostic Criteria for Insomnia, SDQ = Strengths and Difficulties Questionnaire, SDSC = Sleep Disturbance Scale for Children, SEQ‐C = Self‐Efficacy Questionnaire for Children, SWD = sleep–wake disturbances, TAU = treatment as usual, TBI = traumatic brain injury, TOMM = Test of Memory Malingering, TSI = time since injury, UC = usual care, VAS = Visual Analog Scale.

Melatonin treatment protocols in the included trials followed a consistent pattern. In both trials by Barlow et al. (Barlow et al. 2020; Barlow et al. 2021) participants initiated treatment in the subacute phase following injury (mean 38 days post‐injury). Melatonin was administered as a regular nightly treatment, taken sublingually 1 h before bedtime for a fixed 28‐day course rather than on an as‐needed basis. Both trials used a sustained‐release formulation at 3 and 10 mg doses, and treatment continued even if symptoms improved. Outcome assessments were performed at baseline, weekly during treatment, at the end of the 28‐day period, and at follow‐ups at approximately 3 and 6 months post‐injury. Compliance was monitored weekly and overnight urinary 6‐sulphatoxymelatonin (aMT6s) was measured at multiple time points to verify adherence. These protocol details were considered when interpreting heterogeneity across studies.

### 3.2 Other Outcomes

Τen studies assessed TBI‐related neurocognitive symptoms (Barlow et al. 2020; Barlow et al. 2021; Anderson et al. 2021; Barlow et al. 2014; Corwin et al. 2024; Dobney et al. 2017; McNally et al. 2018; Shore et al. 2022; Simpson et al. 2021; Smulligan et al. 2023). Five studies explored educational, emotional, and behavioral functioning and general quality of life (Anderson et al. 2021; Barlow et al. 2014; McNally et al. 2018; Simpson et al. 2021; Tomfohr‐Madsen et al. 2020). Two studies investigated engagement in physical activity (Shore et al. 2022; Simpson et al. 2021). Four studies evaluated intervention quality (Moore et al. 2021; Corwin et al. 2024; Ledoux et al. 2024; Mazzone et al. 2025).

### 3.3 Intervention Effects: A Meta‐Analysis

Values were extracted for (a) total post‐concussion symptomatology (as assessed with the Post‐Concussion Symptom Inventory [PCSI] and self‐reported number of concussion symptoms), (b) cognitive outcomes (as measured with the CNS Vital Signs Neurocognitive Index (Gualtieri and Johnson 2006)), (c) SWD (measured with the ISI (Bastien et al. 2001)), and (d) overall quality of life (measured by Pediatric Quality of Life Inventory (Desai et al. 2014)).

**FIGURE 2:**
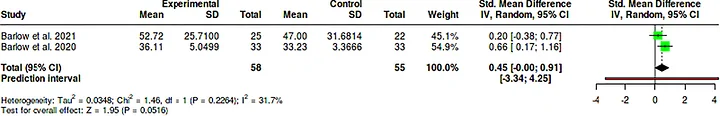
Effects of 3 mg melatonin on persistent post‐concussion symptoms (PPCS). *Note*: Forest plot summarizing standardized mean differences in persistent post‐concussion symptoms for children with TBI who received 3 mg melatonin compared to control groups from Barlow et al. (2020) and Barlow et al. (2021). The overall pooled effect was not statistically significant. Low‐to‐moderate heterogeneity was observed (*I* ^2^ = 31.7%), suggesting relatively consistent effect sizes across studies.

When comparing children that received melatonin (3 mg group; *n* = 58) to controls (*n* = 55), the test for overall effect did not demonstrate a statistically significant effect on overall post‐concussion symptomatology. Using a random effects model with the inverse variance method, the SMD was 0.45 (95% CI = −0.00 to 0.91) (see Figure 2). Low‐to‐moderate heterogeneity was observed (*p* = 0.23) with an *I* ^2^ value of 31.7%, suggesting relatively consistent effect sizes across studies. Although pooled effects did not reach statistical significance, individual studies reported improvements in selected sleep‐related outcomes following melatonin administration (Barlow et al. 2020; Barlow et al. 2021).

**FIGURE 3:**
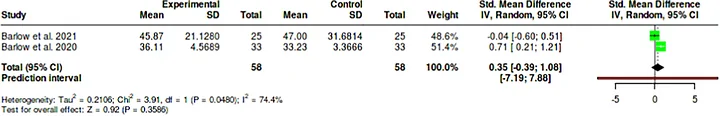
Effects of 10 mg melatonin on persistent post‐concussion symptoms (PPCS). *Note*: Forest plot summarizing standardized mean differences in persistent post‐concussion symptoms for children with TBI who received 10 mg melatonin compared to control groups from Barlow et al. (2020) and Barlow et al. (2021). The overall pooled effect was not statistically significant. Moderate‐to‐high heterogeneity was observed (*I* ^2^ = 74.4%), suggesting variability across studies.

Additionally, when comparing the 10 mg melatonin group (*n* = 58) to controls (*n* = 58), the overall effect again was not statistically significant. The summarized SMD was 0.35 (95% CI = −0.39 to 1.08) (see Figure 3). Heterogeneity remained moderate‐to‐high (*p* = 0.05, *I* ^2^ = 74.4%), suggesting considerable inconsistency in effect sizes.

**FIGURE 4:**
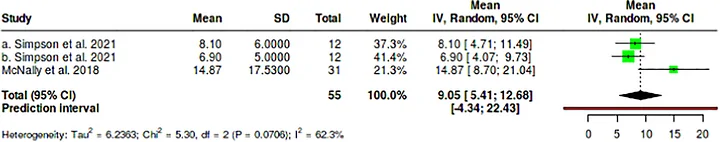
Effects of CBT‐I on persistent post‐concussion symptoms (PPCS) reported by adolescents and parents. *Note*: Forest plot summarizing mean symptom severity scores following CBT‐I interventions from Simpson et al. (2021) and McNally et al. (2018). (a) Adolescent‐reported and (b) parent‐reported outcomes are presented separately. Moderate heterogeneity was observed (*I* ^2^ = 62.3%).

In post‐CBT‐I interventions (*n* = 55), parent and child reports of persistent post‐concussion symptoms (PPCS) were analyzed. The MRAW was 9.05 (95% CI = 5.41–12.68) (see Figure 4). Moderate heterogeneity was observed (*I* ^2^ = 62.3%), suggesting variability in effect magnitude across studies.

**FIGURE 5:**
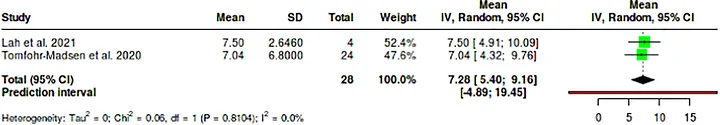
Subjective sleep problem scores following CBT‐I interventions. *Note*: Forest plot summarizing subjective sleep problem scores (Insomnia Severity Index) from Lah et al. (2021) and Tomfohr‐Madsen et al. (2020). Significant heterogeneity was not observed (*I* ^2^ = 0%), indicating that effect sizes across studies were uniform in both magnitude and direction.

For sleep outcomes reported post CBT‐I (*n* = 28), the MRAW was 7.28 (95% CI = 5.40–9.16) (see Figure 5). Significant heterogeneity was not observed (*I* ^2^ = 0%), indicating that effect sizes across studies were uniform in both magnitude and direction.

**FIGURE 6:**
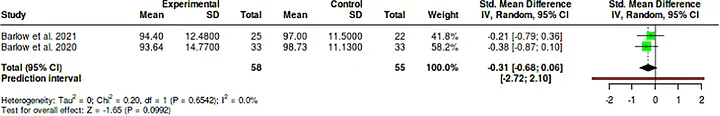
Cognitive outcomes following 3 mg melatonin administration. *Note*: Forest plot displaying standardized mean differences in cognitive outcomes (CNS Vital Signs Neurocognitive Index) comparing 3 mg melatonin and control groups from Barlow et al. (2020) and Barlow et al. (2021). Significant heterogeneity was not observed (*I* ^2^ = 0%), suggesting that effect sizes across studies were uniform in both magnitude and direction.

In terms of cognitive outcomes, when comparing the melatonin 3 mg group (*n* = 58) to controls (*n* = 55), no significant effect was observed. The summarized SMD was −0.31 (95% CI = −0.68 to 0.06) (see Figure 6). Significant heterogeneity was not observed (*I* ^2^ = 0%), suggesting that effect sizes across studies were uniform in both magnitude and direction.

**FIGURE 7:**
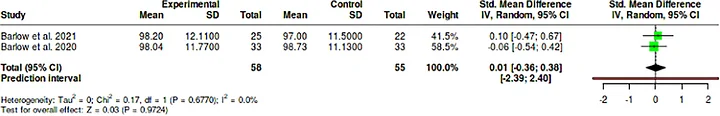
Cognitive outcomes following 10 mg melatonin administration. *Note*: Forest plot displaying standardized mean differences in cognitive outcomes (CNS Vital Signs Neurocognitive Index) comparing 10 mg melatonin and control groups from Barlow et al. (2020) and Barlow et al. (2021). Significant heterogeneity was not observed (*I* ^2^ = 0%), suggesting that effect sizes across studies were uniform in both magnitude and direction.

When comparing the melatonin 10 mg group (*n* = 58) to controls (*n* = 55), no significant effect was observed. The summarized SMD was 0.01 (95% CI = −0.36 to 0.38) (see Figure 7). Significant heterogeneity was not observed (*I* ^2^ = 0%), indicating that effect sizes across studies were uniform in both magnitude and direction.

**FIGURE 8:**
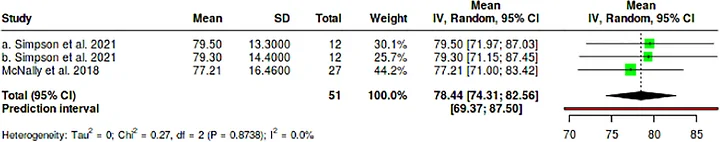
Quality of life outcomes following post‐TBI interventions. *Note*: Forest plot summarizing mean quality of life scores measured with the Pediatric Quality of Life Inventory (PedsQL) following post‐TBI interventions from Simpson et al. (2021) and McNally et al. (2018). (a) Child‐reported and (b) parent‐reported outcomes are presented separately. Significant heterogeneity was not observed (*I* ^2^ = 0%), indicating that effect sizes across studies were consistent in both magnitude and direction.

When evaluating post‐intervention quality of life (*n* = 51), the summarized MRAW was 78.44 (95% CI = 74.31–82.56) (see Figure 8). Significant heterogeneity was not observed (*I* ^2^ = 0%), indicating that effect sizes across studies were consistent in both magnitude and direction.

## 4 Discussion

This systematic review and meta‐analysis evaluated the efficacy of various sleep interventions in children with TBI, focusing on PPCS, cognitive and school functioning, sleep outcomes, and quality of life. The findings partially support these hypotheses, demonstrating effectiveness in certain domains while also revealing variability in treatment responses across interventions.

CBT‐I was found to be effective in reducing PPCS and improving subjective sleep outcomes. However, variability in treatment responses was still observed across some outcomes. CBT‐I produced relatively consistent improvements in subjective sleep problems, whereas actigraphy data were less consistent across trials. This variability may be attributed to baseline sleep problem severity, the extent of injury, or differences in participant engagement. Despite this variation, CBT‐I remains a frontline behavioral intervention for post‐concussion sleep symptoms in pediatric TBI. Pharmacological interventions, particularly melatonin, showed mixed and nonsignificant pooled effects. In the PlayGame randomized trial, children began treatment at an average of 38 days after injury and received sublingual sustained‐release melatonin (3 or 10 mg) nightly for 28 days, taken 1 h before bedtime (Barlow et al. 2020). A secondary analysis focused on participants with clinically significant sleep disturbance and examined short‐term effects during the first 2 weeks of treatment (Barlow et al. 2021). That analysis found modest improvements in subjective sleep problems with 3 mg melatonin, increased total sleep time with both doses, and higher sleep efficiency with 10 mg in the per‐protocol sample (Barlow et al. 2021). These details indicate that melatonin was administered as a regular nightly treatment initiated in the subacute phase (mean 38 days post‐injury), rather than as an acute or as‐needed treatment. The relatively short duration of treatment and initiation in the subacute phase (mean 38 days post‐injury) may partly explain the variability observed across outcomes and studies. Differences in injury severity, individual sleep profiles, and adherence could also contribute to this heterogeneity. Prior research has yielded mixed findings, with some results reporting no significant cognitive benefit of melatonin in children with TBI (McNally et al. 2018). Collectively, the pooled findings of the present review did not demonstrate statistically significant effects of either 3 or 10 mg melatonin on post‐concussion symptomatology or cognitive outcomes, highlighting the need for further investigation into the specific conditions under which melatonin may be most beneficial.

An additional contributor to the high heterogeneity observed across studies may be differences in TBI severity. Severity influences the extent and pattern of neural injury, recovery trajectories, and symptom perception, and has been associated with variation in self‐reported neuropsychiatric and post‐concussion symptom burden (Kim et al. 2023). Differences in case‐mix (mild, moderate, severe) and inconsistent reporting of severity indices (e.g., Glasgow Coma Scale, imaging findings, or clinical classification) across trials could therefore amplify between‐study variability in both subjective and objective sleep outcomes, as well as cognitive and quality‐of‐life measures. This mechanism may partly explain why some interventions (notably CBT‐I) show relatively consistent benefits on self‐report measures while pharmacological trials (e.g., melatonin) report more variable outcomes. Future trials should stratify analyses by injury severity or include severity as a covariate to clarify whether treatment effectiveness differs across TBI subgroups.

The mixed findings observed across melatonin dosage groups contrast with findings from some adult populations, where melatonin has been associated with reductions in sleep latency and improvements in total sleep time (Ferracioli‐Oda et al. 2014). However, the pooled analyses in the present review did not demonstrate statistically significant effects for either 3 or 10 mg melatonin on post‐concussion symptomatology or cognitive outcomes. These findings suggest that treatment response in pediatric TBI may be influenced by developmental differences, injury‐related sleep pathology, or individual variability in melatonin metabolism. Future research should further investigate age‐specific dosing protocols and identify subgroups of children most likely to benefit from melatonin treatment.

Beyond CBT‐I and pharmacological treatments, mindfulness‐based interventions were also examined in this review, although only one study specifically evaluated their effects in pediatric TBI (Ledoux et al. 2024). While the limited data preclude definitive conclusions, preliminary findings suggest that mindfulness techniques may offer an alternative behavioral approach for improving sleep in this population (Ledoux et al. 2024). Mindfulness practices have been shown to modulate physiological arousal and enhance emotional regulation, both of which play a role in sleep problems (Crosswell et al. 2017; Talley and Shelley‐Tremblay 2020). However, their effectiveness in pediatric TBI populations remains underexplored. Future research is needed to determine whether mindfulness interventions can provide sustained benefits for sleep quality and post‐concussion symptomatology.

Although the hypothesis that sleep interventions would uniformly improve quality of life was only partially supported, post‐intervention improvements were observed across several measures. However, the extent of benefit varied considerably, suggesting that the effectiveness of treatment may depend on individual patient characteristics. These findings further highlight the complexity of addressing sleep problems in pediatric TBI and underscore the need for personalized intervention strategies. Heterogeneity varied across melatonin analyses, with lower heterogeneity observed for pooled PPCS outcomes in the 3 mg subgroup, whereas CBT‐I sleep outcomes, cognitive outcomes, and quality‐of‐life outcomes demonstrated relatively consistent effect sizes across studies. Although injury severity was considered a priori as a potential moderator, formal subgroup or meta‐regression analyses by severity were constrained by inconsistent reporting of severity indices across included studies and by small numbers of studies within particular severity strata; therefore, we could not reliably quantify whether intervention effects differed by TBI severity, which remains an important limitation. An additional limitation of the present review is the inclusion of heterogeneous study designs, including RCTs, observational studies, pilot studies, and study protocols. These designs differ in methodological rigor, risk of bias, and capacity to support causal inference. Therefore, although the findings provide a useful overview of available interventions, pooled estimates should be interpreted cautiously, as study design heterogeneity may have contributed to variability across outcomes. Moreover, although the initial hypothesis suggested that sleep interventions would enhance educational outcomes, no eligible studies were identified that directly evaluated the correlation between sleep interventions and school functioning. This gap underscores the need for future research to explore the potential educational benefits of sleep interventions in children with TBI.

In this regard, the current findings are consistent with previous research indicating that CBT‐I is an effective intervention for improving sleep quality in pediatric populations affected by TBI (Moore et al. 2021; Tomfohr‐Madsen et al. 2020). Furthermore, clinical trials have indicated that CBT‐I may also alleviate PPCS (Tomfohr‐Madsen et al. 2020). Aligned with clinical guidelines for treating insomnia in adults (Qaseem et al. 2016), these results indicate that behavioral interventions are essential for improving sleep quality following TBI. Moreover, variability in sleep problems related to comorbidities has been well‐documented (Ekinci et al. 2017; Galli et al. 2022; Schwichtenberg et al. 2022; Halstead et al. 2021; Ogundele and Yemula 2022). This effect reinforces the need for more personalized intervention strategies rather than a one‐size‐fits‐all approach. Future research should focus on identifying specific subgroups of children most likely to benefit from each type of intervention, improving clinical decision‐making and treatment outcomes.

### 4.1 Possible Explanations and Mechanisms

The effects of CBT‐I and melatonin on sleep and cognitive outcomes in children with TBI can be attributed to a combination of neurobiological, behavioral, and methodological factors, particularly those related to sleep assessment. These findings are consistent with prior research on sleep interventions in pediatric populations, particularly among children with neurological conditions (Halstead et al. 2021; Ogundele and Yemula 2022). The efficacy of CBT‐I in alleviating PPCS may be attributed to its ability to improve sleep hygiene, cognitive restructuring, and behavioral regulation (Spencer et al. 2019). Sleep problems following TBI often encompass disruptions in homeostatic and circadian sleep regulation, maladaptive sleep behaviors, and heightened physiological arousal (Rao et al. 2015; Wickwire et al. 2018). Research indicates that structured behavioral interventions, such as CBT‐I, help realign the sleep–wake cycle, enhance self‐regulation, and mitigate insomnia symptoms by targeting dysfunctional sleep beliefs (Thakral et al. 2020). Improved sleep quality also reduces fatigue‐related cognitive impairments and enhances executive function (Williams et al. 2022). By establishing consistent bedtime routines, challenging negative sleep‐related thoughts, and promoting sleep‐supporting behaviors, CBT‐I likely contributes to meaningful improvements in both sleep and cognitive functioning in pediatric TBI.

The mechanisms underlying melatonin's effects are more complex and warrant further investigation. Melatonin regulates sleep–wake cycles by influencing circadian rhythms and facilitating sleep onset, while also exerting potential neuroprotective effects following TBI (Naseem and Parvez 2014). However, the extent to which these mechanisms translate into measurable improvements in pediatric post‐concussion symptomatology or cognitive outcomes remains uncertain. Although individual studies have reported improvements in selected sleep parameters following melatonin administration, the pooled analyses in the present review did not demonstrate statistically significant effects on post‐concussion symptomatology or cognitive outcomes for either 3 or 10 mg melatonin. These findings suggest that dosage alone may not adequately explain treatment response. Individual factors such as melatonin metabolism, injury severity, baseline sleep disturbance, and receptor sensitivity likely influence treatment response. For instance, in pediatric neurological populations, such as autism spectrum disorder, lower doses (1–3 mg) reduce sleep onset latency and increase total sleep time, whereas higher doses (up to 10 mg) may produce greater benefits (Malow et al. 2016). However, whether these effects extend to pediatric TBI remains uncertain. Given the inconsistent findings, further research is needed to determine optimal dosing and identify subgroups most responsive to melatonin. Future studies should investigate biomarkers of melatonin metabolism, evaluate sleep architecture, and consider injury‐specific characteristics to clarify mechanisms underlying differential responses.

A crucial methodological consideration in this meta‐analysis is the distinction between objective (actigraphy) and subjective (self‐report/sleep diaries) sleep measures. While subjective data across several studies indicated improvements in sleep quality (Dobney et al. 2017; McNally et al. 2018; Simpson et al. 2021; Tomfohr‐Madsen et al. 2020; Lah et al. 2021; McCarty et al. 2024), actigraphy data were notably inconsistent. Among six studies employing actigraphy (Barlow et al. 2020; Barlow et al. 2021; Barlow et al. 2014; Corwin et al. 2024; Lah et al. 2021; Tham et al. 2015), only one found significant improvement in sleep efficiency after 10 mg melatonin (Barlow et al. 2020), while another found no group differences (Barlow et al. 2021).

Additionally, actigraphy data from the BCAA study (Corwin et al. 2024) suggested but did not statistically confirm sleep efficiency improvements. Actigraphy objectively assesses sleep parameters, including sleep efficiency, sleep onset latency, and wake after sleep onset (WASO), but does not capture qualitative aspects of sleep, such as sleep satisfaction or perceived restfulness, which are essential indicators of treatment effectiveness (Rhythms 2024). Notably, among the studies using sleep diaries (Tomfohr‐Madsen et al. 2020; Lah et al. 2021), both measured sleep efficiency and latency, but only one assessed WASO (Tomfohr‐Madsen et al. 2020), highlighting inconsistencies in methodology. This variability underscores the limitation of relying solely on objective metrics to evaluate intervention effects.

Discrepancies between objective and subjective sleep measures are well‐documented in pediatric sleep research, particularly in TBI (Tham et al. 2015; Milroy et al. 2008). One potential explanation is sleep misperception, wherein individuals report feeling more rested despite negligible measurable variations in sleep efficiency, a phenomenon observed in post‐concussive and cognitive‐behavioral sleep interventions (McNally et al. 2018). Another contributing factor may be expectation bias, as participants receiving active interventions may anticipate improvements, leading to perceived, but not necessarily physiological, enhancements in sleep quality. This bias is particularly relevant, considering that both CBT‐I (Tomfohr‐Madsen et al. 2020) and pharmacological treatments (Barlow et al. 2020; Barlow et al. 2021) have shown improved subjective outcomes despite inconsistent actigraphy data.

### 4.2 Clinical Implications

The findings of this review have significant clinical implications for addressing sleep problems in children with TBI. CBT‐I has demonstrated efficacy in PPCS and sleep quality, solidifying its role as the primary behavioral intervention for sleep problems in pediatric TBI. Its prioritization in clinical settings is essential for children experiencing persistent sleep difficulties post‐TBI. Based on evidence from both pediatric and adult populations (Moore et al. 2021; Tomfohr‐Madsen et al. 2020), clinical guidelines support its early implementation. Clinicians should incorporate structured CBT‐I programs into rehabilitation plans, emphasizing sleep hygiene, behavioral strategies, and cognitive restructuring to tackle maladaptive sleep beliefs. Furthermore, interventions must also promote patient engagement and adherence, as response to CBT‐I varies depending on injury severity, baseline sleep disturbance, and motivation.

The inconsistent and nonsignificant pooled effects of melatonin suggest that pharmacological interventions should be used judiciously and tailored to individual patient profiles. Although individual studies reported improvements in selected sleep parameters, the pooled analyses did not demonstrate statistically significant effects of either 3 or 10 mg melatonin on post‐concussion symptomatology or cognitive outcomes. Clinicians should therefore consider melatonin metabolism, injury severity, and preexisting sleep patterns when prescribing pharmacological sleep interventions. Additionally, monitoring both subjective and objective (actigraphy) outcomes is essential, as discrepancies between subjective reports and actigraphy data highlight the need for multi‐method assessment rather than reliance on self‐report alone (Barlow et al. 2020; Barlow et al. 2021).

In addition to CBT‐I, mindfulness‐based interventions have shown promise, yet remain underexplored in pediatric TBI. Given their ability to modulate physiological arousal and address emotional dysregulation, mindfulness techniques may serve as a valuable adjunct therapy, particularly in children with co‐occurring anxiety or mood disorders (Zoogman et al. 2015; Borquist‐Conlon et al. 2019). Future clinical practice should consider integrating mindfulness into sleep programs, especially for patients showing limited response to CBT‐I or pharmacological treatments. Further research is needed to determine its efficacy in this population.

An additional underexplored area in pediatric TBI is the role of physical activity in sleep rehabilitation. Evidence suggests that structured exercise can enhance sleep quality and cognitive function in pediatric and neurological populations (Liu et al. 2024; Zhang et al. 2024; Karelina et al. 2021; Herrera and Leon‐Rojas 2024), though its benefits following TBI remain unclear. This review identified limited studies employing physical activity‐based interventions, such as virtual rehabilitation programs (Anderson et al. 2021; Simpson et al. 2021), but evidence regarding their effectiveness for sleep remains limited. Sleep problems in children recovering from TBI often co‐occur with neurocognitive and emotional dysfunction (Dobney et al. 2017; McNally et al. 2018), and emerging evidence suggests that structured exercise may contribute to improvements in post‐concussive symptoms and quality of life (Anderson et al. 2021; Simpson et al. 2021). Exercise has been shown to promote neuroplasticity, reduce fatigue, and support mood regulation in children with brain injuries (Karelina et al. 2021; Herrera and Leon‐Rojas 2024), which are essential components of sleep rehabilitation. Future research should investigate the efficacy of structured exercise programs as a non‐pharmacological intervention for sleep problems in pediatric TBI. Key focus areas should include identifying the optimal exercise intensity, duration, and modality to maximize its therapeutic effects on sleep and overall neurocognitive recovery. Despite initial hypotheses suggesting benefits for school functioning, no studies to date have specifically investigated the impact of sleep interventions on educational outcomes. Given that sleep problems contribute to cognitive dysfunction, attentional deficits, and academic struggles, future clinical practice should incorporate school‐based screenings and collaborate closely with educators and healthcare providers to develop sleep‐informed academic accommodations. This issue is particularly relevant for children recovering from TBI, who may experience prolonged school absences or struggle to reintegrate into educational settings. Considering the complexity of pediatric TBI and sleep problems, a multidisciplinary approach is essential for integrating neuropsychologists, sleep specialists, rehabilitation therapists, and pediatricians. Sleep problems in TBI patients often co‐occur with cognitive deficits, emotional dysregulation, and functional impairments, requiring comprehensive intervention strategies that address both sleep and neurocognitive recovery (Weber et al. 2013; Moore et al. 2021). Long‐term follow‐up evaluations should be a standard element of sleep intervention programs, ensuring that treatment effects are sustained and appropriately adjusted over time.

## 5 Conclusions

This systematic review and meta‐analysis comprehensively evaluated sleep interventions for children with TBI, underscoring the potential benefits and limitations of current treatment approaches. The findings indicate that CBT‐I emerged as the most consistently effective intervention for improving PPCS and sleep outcomes. Nevertheless, heterogeneity in individual responses highlights the need for tailored approaches based on injury severity and preexisting sleep problems. Pharmacological treatments, especially melatonin, produced mixed outcomes across studies and pooled analyses. Although melatonin demonstrated some improvements across selected subjective and cognitive measures in individual studies (Barlow et al. 2020; Barlow et al. 2021), pooled meta‐analytic findings did not demonstrate statistically significant overall effects and should therefore be interpreted cautiously. Variability across studies may reflect differences in injury severity, participant age, intervention timing, and methodological design rather than a consistent dose‐response effect. Future research should further investigate optimal dosing strategies and identify subgroups most likely to benefit from melatonin treatment. Furthermore, actigraphy data revealed discrepancies between objective and subjective sleep improvements, underscoring the need for multi‐method assessments in future research. Emerging interventions, including mindfulness‐based therapies and structured physical activities, offer viable non‐pharmacological alternatives. Despite limitations in the available data, these methodologies merit additional investigation owing to their prospective contributions to the modulation of physiological arousal, neuroplasticity, and emotional regulation. Given the significant prevalence of co‐occurring cognitive and emotional dysfunction in pediatric TBI, interventions targeting both sleep and neurocognitive recovery should be prioritized in clinical practice.

The review also identified significant research gaps, notably the absence of studies exploring the effects of sleep interventions on academic performance. Addressing these gaps through meticulously designed RCTs will be critical in refining treatment strategies and optimizing recovery outcomes for pediatric patients suffering from TBI. The inclusion of heterogeneous study designs, including RCTs, observational studies, pilot studies, and study protocols, may have influenced the consistency and interpretability of pooled findings. Differences in methodological rigor and risk of bias across study designs limit the strength of causal inference and should be considered when interpreting the results. Future research should prioritize the development of personalized treatment models that integrate behavioral, pharmacological, and rehabilitative approaches, thereby accounting for individual differences. A multidisciplinary approach incorporating neuropsychologists, sleep specialists, rehabilitation therapists, and pediatricians is imperative to effectively address the intricate relationship between sleep problems and broader neurocognitive impairments.

## Author Contributions

**K. Lofitou**: conceptualization, investigation, writing – original draft, methodology, validation, visualization, writing – review and editing, software, formal analysis, project administration, data curation, supervision, resources. **E. P. Kouki**: methodology, writing – review and editing, writing – original draft, software, formal analysis, data curation. **C. Gavriel**:writing – review and editing, formal analysis, methodology, data curation. **H. Achilleos**: methodology, writing – review and editing, formal analysis, data curation. **V. Themistokleous**: writing – review and editing, methodology, formal analysis, data curation. **P. Bargiotas**: writing – review and editing, supervision. **J. Haarbauer‐Krupa**: writing – review and editing, supervision. **E. Paraskevopoulos**: writing – review and editing, supervision. **F. Constantinidou**: supervision, resources, project administration, writing – review and editing, visualization.

## Funding

This work was supported by the Center for Applied Neuroscience, University of Cyprus.

## Ethics Statement

This study was conducted as part of the doctoral research of the first author (K. Lofitou) and received ethical approval from the Cyprus National Bioethics Committee and the State Health Services Organization (SHSO). As this review involved secondary analysis of previously published studies, no additional participant consent was required.

## Conflicts of Interest

The authors declare no conflicts of interest.

## Supporting information




**Supplementary Material**: brb371556‐sup‐0001‐SuppMat.docx

## Data Availability

The PRISMA 2020 checklist, the full search strategies for all databases, and the quality assessment are provided in the Supporting Information and are available in the Open Science Framework (OSF) at: https://osf.io/4jhmr/?view_only=967c2c0a3201490eb722226adfbb8e6a.
