# Supplementary material for: Sleep–Wake Disturbances in Pediatric Traumatic Brain Injury: A Systematic Review and Meta‐Analysis of Interventions
Source: Brain Behav. 2026 Sep 21;16(9):e71556. doi: 10.1002/brb3.71556 (PMC13594777; doi:10.1002/brb3.71556)
Supplement: Supplementary file 1 [file BRB3-16-e71556-s001.docx]

*Search strategy*

In May 2024, a systematic search was performed across PubMed, Web of Science, and ScienceDirect to identify relevant studies on sleep interventions for children and adolescents with TBI. The search was limited to research studies published in English involving participants aged 0-17 and 11 months. No date restrictions were applied, allowing the inclusion of studies from 1998 to 2024.

The search strategy included combinations of "Traumatic Brain Injury" OR "TBI" AND "Children" OR "Pediatrics" AND "Sleep Intervention," along with specific interventions such as "Cognitive Behavioral Therapy (CBT)," "Mindfulness," "Sleep Hygiene Education," "Pharmacological Treatments" (melatonin or other sleep-related drugs), and "Family Therapy." Additional search terms related to cognitive functioning, educational outcomes, and school performance were included. Boolean operators and Medical Subject Headings (MeSH) terms were used, where applicable, to refine search results. The PubMed search included queries such as (((Traumatic Brain Injury) AND (Children)) AND (Sleep Intervention)) AND (CBT), resulting in 334 studies. In comparison, similar searches on the Web of Science retrieved 75 studies, and ScienceDirect identified 2532 studies. Each database was searched using tailored strategies, with “ALL” field searches in Web of Science and “OR/AND” operators in ScienceDirect to ensure comprehensive coverage.

To ensure the inclusion of the most recent literature, a follow-up search was conducted in February 2025 to identify newly published studies meeting the eligibility criteria. Additionally, reference lists of identified systematic reviews and included empirical studies were manually screened (ancestry search) to identify additional relevant articles. The PRISMA 2020 checklist and the full search strategies for all databases are available on the Open Science Framework (OSF): <https://osf.io/4jhmr/?view_only=967c2c0a3201490eb722226adfbb8e6a>.

*Quality assessment*

The quality of included studies was assessed using standardized appraisal tools based on study design appropriate for each intervention type. Case studies (n=3) were evaluated using the quantitative Critical Appraisal Skills Programme (CASP) scale^28^. Randomized controlled trials (n=4) were assessed using the CASP RCT assessment scale. Intervention protocols (n=6) were evaluated using The Template for Intervention Description and Replication (TIDieR) checklist^29^, and observational studies (n=6) were evaluated with the STROBE Statement^30^ (Strengthening the Reporting of Observational Studies in Epidemiology).

Studies with a reporting percentage above 70%, as assessed by both reviewers in the appropriate quality assessment tool, were considered sufficient for inclusion in the review. Studies that fell below this threshold were further examined, and if they exhibited substantial methodological limitations, they were discussed by the reviewers to determine final inclusion.
